# Editor's Letter Box

**Published:** 1891-06-06

**Authors:** 


Sir,—In view of the many deaths from bronchitis and pneumonia following on influenza in the present epidemic and apropos of the admirable annotation in The Hospital, permit me to say that I have had some experience in the treatment of influenza. I have never seen a death from influenza but once, and that in a patient the subject of advanced phthisis. I believe the cause of most deaths has been from the use of such drugs as antipyrin and its congeners, which powerfully liquify the blood and precipitate and even bring on bronchial and pulmonary mischief by increasing effusion in the pulmonary tract. I use peripheral vascular stimulants as Sp. Ether nit. and Tr. Opii to relieve the pain, and increasing doses of iron. In this way the temperature soon falls to normal, the pain is relieved, if there is any cough it disappears. Recovery is rapid, convalescence is not prolonged. I believe that iron should be our sheet anchor. I believe this method guards against any chest complication. I shall be glad to give further particulars if any of your readers desire them.—Yours faithfully, Enthusiast.

May 16th, 1891.

